# Prognostic Value of a Low-Cost LDH–Hemoglobin–Albumin Biomarker Panel in Acute Heart Failure: A Real-World Cohort from a Resource-Limited Setting

**DOI:** 10.3390/biomedicines14030704

**Published:** 2026-03-18

**Authors:** Can Baba Arin

**Affiliations:** Dr. Siyami Ersek Thoracic and Cardiovascular Surgery Training and Research Hospital Istanbul, Istanbul 34668, Türkiye; canbaba.arin@saglik.gov.tr

**Keywords:** acute heart failure, lactate dehydrogenase, low-cost biomarkers, in-hospital mortality, risk stratification, resource-limited settings

## Abstract

**Background**: In many low- and middle-income countries, access to advanced cardiac biomarkers such as B-type natriuretic peptide (BNP) and NT-pro BNP remains limited, posing challenges for early risk stratification in patients hospitalized with acute heart failure (AHF). Identifying simple, inexpensive, and universally available laboratory markers with prognostic value is of practical clinical importance. **Methods**: Consecutive patients (≥18 years) hospitalized with acute heart failure (AHF) between May 2022 and November 2024 were retrospectively analyzed. After exclusion of patients with incomplete outcome data, in-hospital mortality was assessed using logistic regression analysis. Hemoglobin, serum albumin, lactate dehydrogenase (LDH), neutrophil-to-lymphocyte ratio (NLR), and the C-reactive protein–albumin–lymphocyte (CALLY) index were evaluated as potential predictors of in-hospital mortality. **Results**: A total of 211 patients were included in the mortality analysis, with an in-hospital mortality rate of 10.0%. Patients were stratified by anemia and hypoalbuminemia status, revealing significant differences in unadjusted mortality rates across groups (*p* = 0.04). However, after adjustment for age, sex, and chronic kidney disease, the prognostic impact of anemia and hypoalbuminemia was attenuated. Elevated LDH levels remained independently associated with in-hospital mortality (adjusted odds ratio 2.84, 95% confidence interval 1.01–8.02). Higher NLR values and lower CALLY index levels showed nonsignificant trends toward adverse outcomes. **Conclusions**: In this real-world cohort from a resource-limited setting, LDH emerged as a practical and independent predictor of in-hospital mortality in patients with AHF. When access to natriuretic peptides is limited, LDH—supported by routinely available laboratory parameters—may assist early risk stratification and clinical decision-making.

## 1. Introduction

Acute heart failure (AHF) remains a major global health problem and continues to be associated with substantial morbidity and mortality despite advances in pharmacological and device-based therapies [1,2,3]. These outcomes are disproportionately worse in low- and middle-income countries, where delayed presentation, limited access to specialized care, and restricted diagnostic resources complicate optimal management [4].

Risk stratification plays a central role in the early management of AHF. In contemporary practice, natriuretic peptides such as BNP and NT-pro BNP are widely used to aid diagnosis and prognostication [5]. However, in many healthcare systems—including several regions in Africa, the Middle East, and parts of Eastern Europe—routine measurement of these biomarkers is often unavailable due to financial and infrastructural constraints [6]. In such settings, clinicians must rely on basic laboratory parameters that are readily accessible and inexpensive.

Anemia and hypoalbuminemia are frequently observed in patients hospitalized with AHF and reflect distinct but overlapping pathophysiological mechanisms. Anemia may exacerbate myocardial hypoxia and neurohormonal activation, whereas hypoalbuminemia is associated with systemic inflammation, malnutrition, venous congestion, and impaired hepatic synthesis [7,8,9,10,11]. Although both conditions have been linked to adverse outcomes in heart failure, their independent and combined prognostic value in acute settings remains inconsistent across studies [12,13,14,15].

Recently, attention has shifted toward low-cost inflammatory and nutritional biomarkers that are universally available, even in resource-limited hospitals. Among these, the neutrophil-to-lymphocyte ratio (NLR) and the C-reactive protein–albumin–lymphocyte (CALLY) index have been proposed as markers of systemic inflammation and immune dysregulation in cardiovascular disease [16,17,18,19,20]. Lactate dehydrogenase (LDH), a marker of tissue hypoxia and cellular injury, has also emerged as a potential indicator of short-term mortality in AHF, particularly in patients with hemodynamic compromise [21,22,23,24].

Importantly, limitations in access to advanced biomarkers are not confined to a single geographic region. Similar challenges are encountered in several middle-income countries, where pragmatic and reproducible prognostic tools remain clinically relevant [25]. Identifying simple laboratory parameters with incremental prognostic value may therefore contribute to more equitable heart failure care across diverse healthcare settings.

Accordingly, this study aimed to evaluate the prognostic significance of commonly available laboratory markers—including hemoglobin, albumin, LDH, NLR, and the CALLY index—for predicting in-hospital mortality among patients hospitalized with AHF in a real-world, resource-limited setting.

Acute heart failure should not be viewed as an isolated entity, but rather as a critical phase within the continuum of chronic heart failure [1,2]. Episodes of acute decompensation often reflect progression of underlying chronic disease and are associated with substantial short- and long-term mortality [1,2,3]. Early risk stratification during hospitalization may therefore influence not only in-hospital management but also subsequent chronic heart failure care [2]. In this context, identifying pragmatic and widely available biomarkers with prognostic value during acute presentations remains highly relevant to the broader field of chronic heart failure [3].

## 2. Materials and Methods

### 2.1. Study Design and Setting

This retrospective observational study was conducted at Mogadishu Somali–Turkey Recep Tayyip Erdoğan Training and Research Hospital, one of the largest tertiary referral centers in Somalia. The hospital serves as a major regional center in are source-limited healthcare environment, where access to advanced cardiac biomarkers such as B-type natriuretic peptide (BNP) or NT-pro BNP is not routinely available due to laboratory and financial constraints.

The study protocol was approved by the local Institutional Review Board (Approval No: MSTH/20379, Date: 7 December 2024). Given the retrospective design and use of anonymized data, the requirement for informed consent was waived. All procedures were conducted in accordance with the Declaration of Helsinki.

### 2.2. Study Population

Consecutive adult patients (≥18 years) hospitalized with a primary diagnosis of acute heart failure (AHF) between May 2022 and November 2024 were screened for eligibility. Acute heart failure was diagnosed based on acute onset or worsening of heart failure symptoms requiring hospital admission, supported by clinical assessment and imaging findings consistent with congestion.

Patients were excluded if:

1. In-hospital outcome data were unavailable (*n*= 9);
2. They presented with severe concomitant infections;
3. They had acute coronary syndromes requiring immediate revascularization;
4. They had active malignancy at the time of admission.

**Figure 1:**
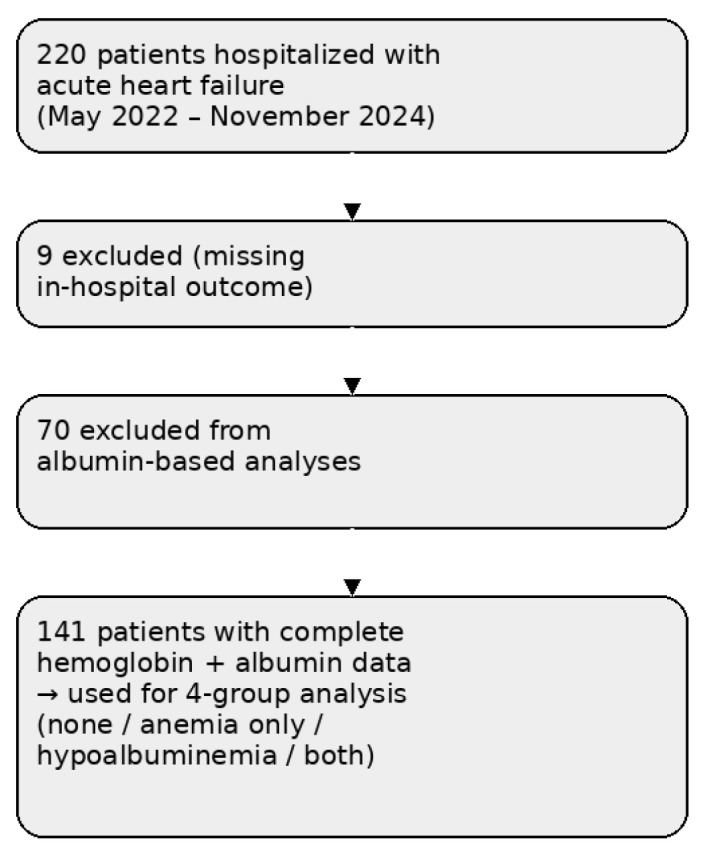
Flow diagram illustrating patient selection for the study. A total of 220 consecutive patients hospitalized with acute heart failure between May 2022 and November 2024 were screened. After exclusion of patients with missing in-hospital outcome data (*n* = 9), 211 patients were included in the primary mortality analysis. Subgroup analyses based on hemoglobin and albumin levels were performed in 141 patients with complete data.

After applying these criteria, 211 patients were included in the final analysis of in-hospital mortality. Subgroup analyses evaluating anemia and hypoalbuminemia were restricted to patients with complete hemoglobin and serum albumin measurements at admission (*n* = 141) to avoid imputation-related bias. The patient selection process is summarized in Figure 1.

### 2.3. Clinical and Laboratory Data Collection

Demographic characteristics, medical history, comorbid conditions, vital signs, and laboratory data were obtained from electronic medical records. All laboratory measurements were derived from blood samples collected within the first 24 h of hospital admission as part of routine clinical care. For patients with multiple measurements during this period, the first available laboratory values were used for analysis.

Laboratory parameters included hemoglobin, serum albumin, lactate dehydrogenase (LDH), C-reactive protein (CRP), total leukocyte count, neutrophil count, and lymphocyte count. Due to local laboratory limitations, natriuretic peptide measurements (BNP or NT-pro BNP) were not available for assessment.

### 2.4. Definitions

Anemia was defined according to World Health Organization criteria as hemoglobin < 13 g/dL in men and <12 g/dL in women. Hypoalbuminemia was defined as a serum albumin concentration < 3.5 g/dL at admission.

The neutrophil-to-lymphocyte ratio (NLR) was calculated by dividing the absolute neutrophil count by the absolute lymphocyte count. The C-reactive protein–albumin–lymphocyte (CALLY) index was calculated using the following formula, as previously described:

CALLY = (albumin × lymphocyte count)/CRP.

The LDH cut-off value (>225 U/L) was defined according to the upper limit of normal provided by the institutional laboratory reference range.

Patients were categorized into four groups based on baseline hemoglobin and albumin levels:

(1) Neither anemia nor hypoalbuminemia;
(2) Anemia only;
(3) Hypoalbuminemia only;
(4) Both anemia and hypoalbuminemia.

### 2.5. Outcome Measure

The primary outcome of the study was all-cause in-hospital mortality, defined as death from any cause during the index hospitalization.

### 2.6. Statistical Analysis

Statistical analyses were performed using SPSS software version 25.0 (IBM Corp., Armonk, NY, USA). Continuous variables were tested for normality using the Kolmogorov–Smirnov test and are presented as mean ± standard deviation or median (interquartile range), as appropriate. Categorical variables are expressed as frequencies and percentages.

Between-group comparisons were conducted using Student’s *t*-test or Mann–Whitney U test for continuous variables and chi-square test or Fisher’s exact test for categorical variables, as appropriate.

Univariate logistic regression analyses were initially performed to identify potential predictors of in-hospital mortality. Variables with clinical relevance or a univariate *p* value < 0.10 were entered into multivariable logistic regression models. Multivariable analyses were adjusted for age, sex, and chronic kidney disease. To minimize model overfitting, the number of covariates included in the final model was limited in relation to the number of outcome events. Results are reported as odds ratios (ORs) with 95% confidence intervals (CIs).

A two-sided *p* value < 0.05 was considered statistically significant.

## 3. Results

### 3.1. Baseline Characteristics

A total of 220 patients hospitalized with acute heart failure were included in the study. The mean age of the cohort was 59.5 ± 14.7 years, and 62.7% of patients were male. Chronic kidney disease was present in 13.2% of the population. Overall in-hospital mortality was 10.0%.

Anemia and hypoalbuminemia were common, observed in 68.7% and 54.6% of patients, respectively. Baseline demographic, clinical, and laboratory characteristics of the study population are summarized in Table 1.

Mortality Across Biomarker Subgroups. Overall in-hospital mortality was 10.0% (*n* = 21). Mortality differed significantly across hemoglobin–albumin subgroups (Table 2).

### 3.2. Prognostic Value of Hemoglobin and Albumin

In univariate logistic regression analysis, both anemia and hypoalbuminemia were associated with higher in-hospital mortality. However, after adjustment in the multivariable logistic regression model, neither anemia nor hypoalbuminemia retained independent prognostic significance for in-hospital mortality. These findings indicate that although anemia and hypoalbuminemia are associated with adverse outcomes at a crude level, their prognostic effect is largely attenuated after accounting for relevant clinical covariates.

### 3.3. Prognostic Value of LDH, NLR, and the CALLY Index

Elevated lactate dehydrogenase (LDH) levels were significantly more frequent among patients who died during hospitalization compared with survivors (*p* < 0.01). In multivariable logistic regression analysis, LDH remained the only laboratory marker independently associated with in-hospital mortality (adjusted odds ratio 2.84, 95%confidence interval 1.01–8.02; *p* = 0.048).

In contrast, inflammatory–nutritional indices showed only nonsignificant trends toward increased mortality. Patients with a high neutrophil-to-lymphocyte ratio (>3.58) and those with a low C-reactive protein–albumin–lymphocyte (CALLY) index (≤0.03) tended to have worse outcomes; however, these associations did not reach statistical significance (*p* = 0.09 and *p* = 0.11, respectively).

### 3.4. Multivariable Model Performance

**Figure 2:**
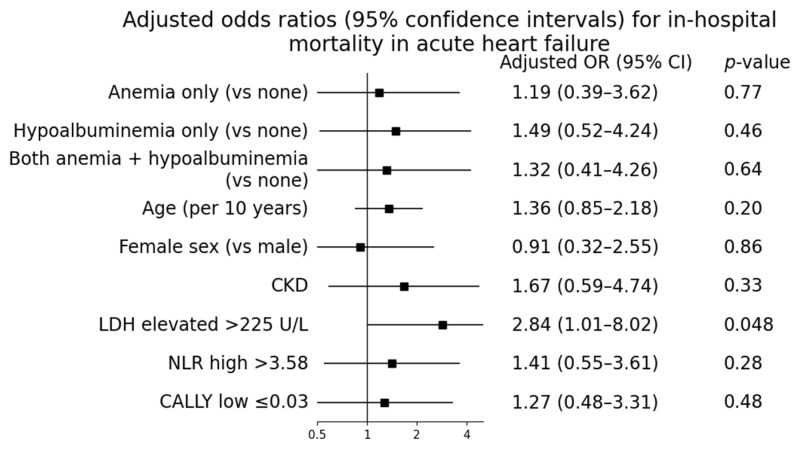
Multivariable logistic regression analysis for in-hospital mortality. Forest plot showing adjusted odds ratios and 95% confidence intervals for predictors of in-hospital mortality derived from the multivariable logistic regression model. The model was adjusted for age, sex, chronic kidney disease, hemoglobin, albumin, lactate dehydrogenase (LDH), neutrophil-to-lymphocyte ratio (NLR), and the C-reactive protein–albumin–lymphocyte (CALLY) index. LDH was the only laboratory parameter independently associated with in-hospital mortality.

**Figure 3:**
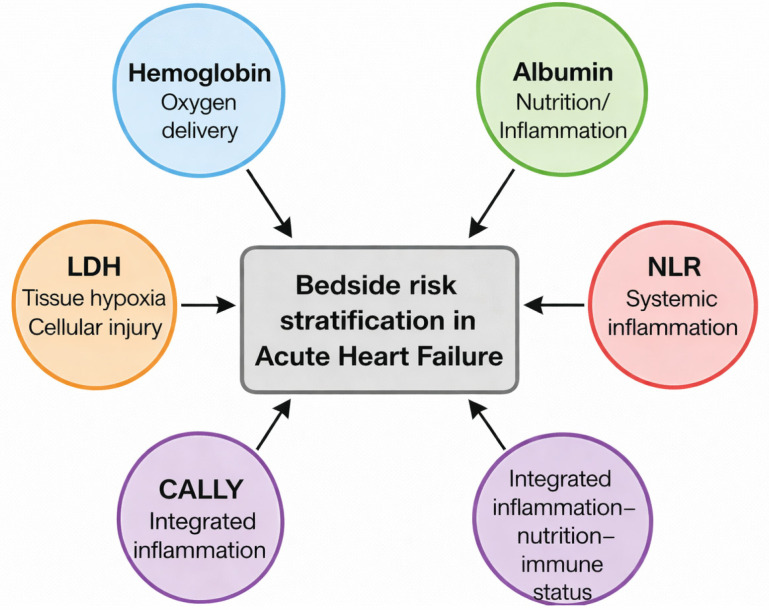
Conceptual schematic of the accessible biomarker panel. Conceptual schematic illustrating complementary biological domains represented by routinely available biomarkers, including hemoglobin (oxygen delivery), albumin (nutrition and inflammation), lactate dehydrogenase (tissue hypoxia and cellular injury), neutrophil-to-lymphocyte ratio (systemic inflammation), and the C-reactive protein–albumin–lymphocyte (CALLY) index (integrated inflammation–nutrition–immune status), used for bedside risk stratification in acute heart failure.

The final multivariable logistic regression model demonstrated modest discrimination (AUC = 0.637). The receiver operating characteristic (ROC) curve of the multivariable model is provided in Supplementary Figure S1. After adjustment for age, sex, chronic kidney disease, hemoglobin, albumin, neutrophil-to-lymphocyte ratio, and the CALLY index, LDH remained the only laboratory parameter independently associated with in-hospital mortality. Multivariable logistic regression results are presented in Table 3 and visualized in Figure 2. The conceptual framework summarizing the complementary biological domains represented by the accessible biomarker panel is illustrated in Figure 3.

## 4. Highlights

- Elevated LDH was independently associated with in-hospital mortality in acute heart failure.
- Anemia and hypoalbuminemia were associated with higher crude mortality but not independent after adjustment.
- Low-cost laboratory biomarkers may support practical risk stratification.
- The study reflects real-world challenges in resource-limited settings.

## 5. Discussion

In this real-world cohort of patients hospitalized with acute heart failure (AHF) in are source-limited setting, lactate dehydrogenase (LDH) was independently associated with in-hospital mortality. These findings suggest that LDH may serve as a practical marker for short-term risk stratification in settings where access to advanced cardiac biomarkers is limited [23,26].

In contrast, LDH remained independently associated with in-hospital mortality after adjustment for age, sex, chronic kidney disease, and other laboratory parameters. LDH is a biologically plausible marker of tissue hypoxia and cellular injury, which are central features of acute heart failure decompensation [23,26]. Elevated LDH levels may reflect systemic hypoperfusion and multiorgan stress [23,26].

Notably, this association was observed in the absence of natriuretic peptide testing, underscoring the potential relevance of LDH in low-resource settings where guideline-recommended biomarkers are often unavailable [27,28]. Although systemic inflammation and immune–nutritional imbalance contribute to heart failure progression [23,29,30,31,32,33,34], their incremental prognostic value for in-hospital risk prediction appeared limited in this cohort.

Anemia and hypoalbuminemia are common in patients with AHF and have been associated with adverse outcomes in prior studies [5,6,7,8,9,10,11,12]. In our analysis, their associations with mortality were attenuated after multivariable adjustment, suggesting that they may reflect overall disease severity and comorbidity burden rather than independent drivers of short-term mortality [12,13,14,15].

## 6. Limitations

Several limitations of this study should be acknowledged. First, its retrospective and single-center design limits causal inference and generalizability. Second, the absence of natriuretic peptide measurements precluded direct comparison with guideline-recommended biomarkers; however, this limitation reflects the real-world constraints faced in many low-resource healthcare systems. Third, the number of in-hospital deaths was relatively modest, which may have limited statistical power and contributed to wide confidence intervals. Finally, long-term outcomes were not assessed, restricting conclusions to short-term prognosis. Given the limited number of outcome events, the multivariable model may be vulnerable to overfitting.

## 7. Conclusions

In this real-world cohort from a resource-limited setting, lactate dehydrogenase emerged as the only laboratory marker independently associated with in-hospital mortality in patients with acute heart failure. Although anemia and hypoalbuminemia were common and associated with crude mortality, they did not retain independent prognostic significance after adjustment for clinical factors. These findings suggest that LDH, a low-cost and universally available biomarker, may provide a practical tool for early risk stratification when access to advanced cardiac biomarkers is limited. Prospective, multicenter studies are warranted to validate these observations.

## Figures and Tables

**Table 1:** Baseline demographic, clinical, and laboratory characteristics of the study population.

Variable	Overall Cohort (*n* = 220)
Age, years	59.5 ± 14.7
Male sex, *n* (%)	138 (62.7)
Diabetes mellitus, *n* (%)	129 (58.6)
Hypertension, *n* (%)	118 (53.6)
Chronic kidney disease, *n* (%)	29 (13.2)
Ischemic heart disease, *n* (%)	11 (5.0)
Hemoglobin, g/dL	11.7 (IQR 10.0–13.3)
Serum albumin, g/dL	3.0 (IQR 2.4–3.8)
Lactate dehydrogenase, U/L	545 (IQR 200–1230)
C-reactive protein, mg/L	45.0 (IQR 17.0–99.0)
Neutrophil-to-lymphocyte ratio	3.6 (IQR 2.3–6.9)

Data are presented as mean ± standard deviation or median (interquartile range), unless otherwise indicated. CKD: chronic kidney disease; LDH: lactate dehydrogenase; CRP: C-reactive protein. LDH values are presented as raw admission measurements; dichotomization for regression analysis was performed using a predefined cut-off.

**Table 2:** In-hospital mortality according to anemia and hypoalbuminemia status.

Hemoglobin–Albumin Subgroup	*n*	In-Hospital Mortality, *n* (%)
Neither anemia nor hypoalbuminemia	38	2 (5.3)
Anemia only	32	6 (18.8)
Hypoalbuminemia only	34	8 (23.5)
Both Anemia and Hypoalbuminemia	37	5 (14.8)
**Total**	141	21 (14.9)

*p* value (overall comparison): 0.04. Data are presented as number (percentage).

**Table 3:** Multivariable logistic regression analysis for predictors of in-hospital mortality.

Variable	Adjusted OR	95% Confidence Interval	*p* Value
Age (per year increase)	1.03	0.99–1.07	0.11
Male sex	1.21	0.48–3.05	0.68
Chronic kidney disease	1.89	0.72–4.96	0.20
Anemia	1.42	0.54–3.71	0.47
Hypoalbuminemia	1.67	0.63–4.45	0.30
Lactate dehydrogenase	2.84	1.01–8.02	0.048
Neutrophil-to-lymphocyte ratio > 3.58	1.96	0.91–4.24	0.09
CALLY index ≤0.03	1.88	0.85–4.13	0.11

Odds ratios were adjusted for age, sex, and chronic kidney disease. OR: odds ratio; CI: confidence interval; CKD: chronic kidney disease; LDH: lactate dehydrogenase. LDH > 225 U/L.

## Data Availability

The data supporting the findings of this study are not publicly available due to patient privacy concerns and institutional restrictions. De-identified data may be made available from the corresponding author upon reasonable request and with appropriate institutional approvals.

## Supplementary Materials

The following supporting information can be downloaded at: https://www.mdpi.com/article/10.3390/biomedicines14030704/s1. Figure S1. Forest plot of multivariable logistic regression analysis for predictors of in-hospital mortality.
