# Supplementary figures and images for: Prognostic Value of a Low-Cost LDH–Hemoglobin–Albumin Biomarker Panel in Acute Heart Failure: A Real-World Cohort from a Resource-Limited Setting
Source: Biomedicines. 2026 Mar 18;14(3):704. doi: 10.3390/biomedicines14030704 (PMC13024537; doi:10.3390/biomedicines14030704)

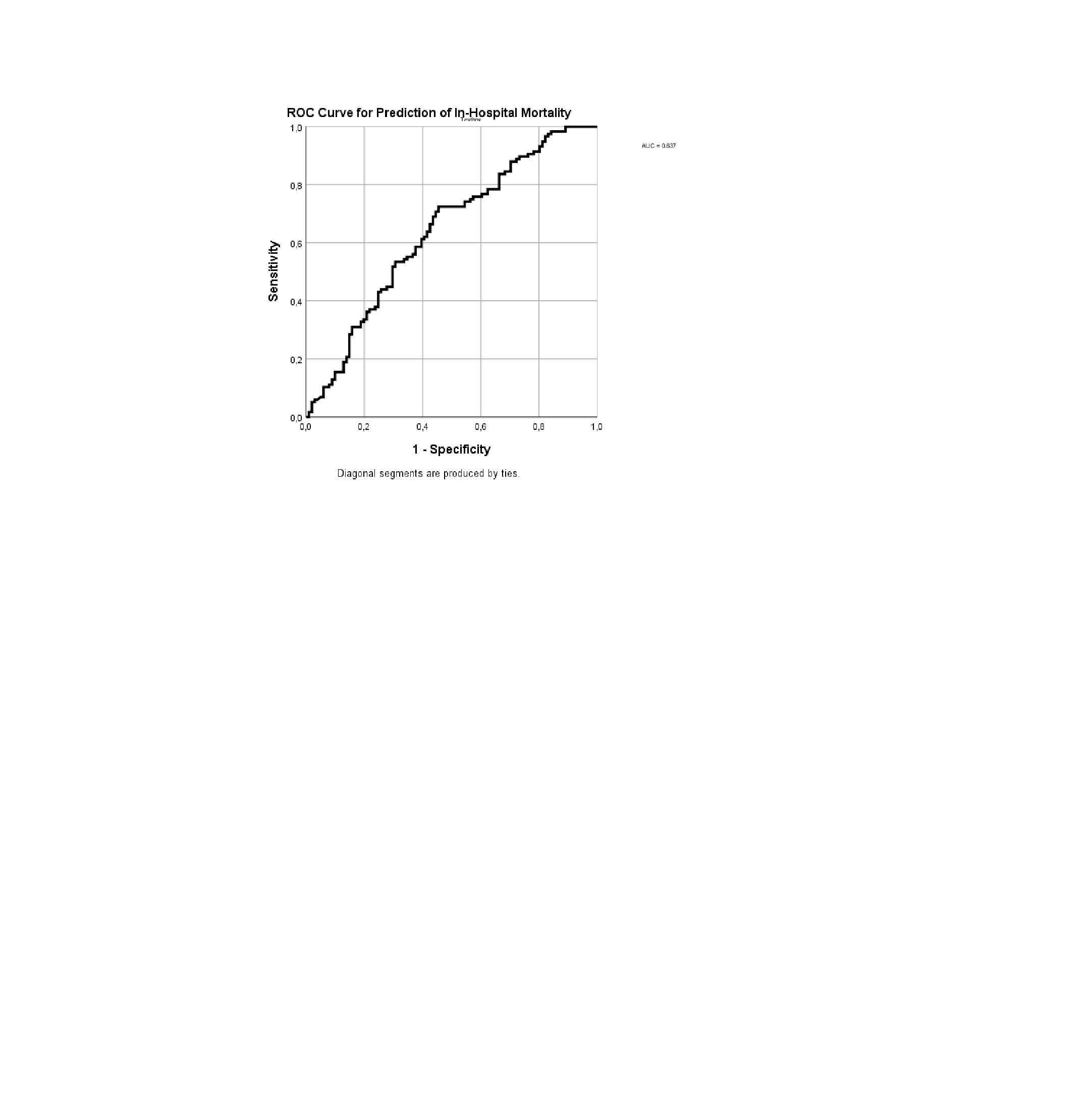

Supplement: Supplementary file 1 [file biomedicines-14-00704-s001.zip › biomedicines-4161299-supplementary.png]
